# The Prevention and Reactivation Care Program: intervention fidelity matters

**DOI:** 10.1186/1472-6963-13-29

**Published:** 2013-01-26

**Authors:** Annemarie JBM de Vos, Ton JEM Bakker, Paul L de Vreede, Jeroen DH van Wijngaarden, Ewout W Steyerberg, Johan P Mackenbach, Anna P Nieboer

**Affiliations:** 1 Institute of Health Policy and Management, Erasmus University Rotterdam, P.O. Box 1738, 3000, DR, Rotterdam, The Netherlands; 2 Department R & D, Argos Zorggroep, P.O. Box 4023, 3102, GA, Schiedam, The Netherlands; 3 Department of Public Health, Erasmus Medical Centre Rotterdam, P.O. Box 2040, 3000, CA, Rotterdam, The Netherlands

**Keywords:** Geriatric care intervention, Intervention fidelity, Moderating factors

## Abstract

**Background:**

The Prevention and Reactivation Care Program (PReCaP) entails an innovative multidisciplinary, integrated and goal oriented approach aimed at reducing hospital related functional decline among elderly patients. Despite calls for process evaluation as an essential component of clinical trials in the geriatric care field, studies assessing fidelity lag behind the number of effect studies. The threefold purpose of this study was (1) to systematically assess intervention fidelity of the hospital phase of the PReCaP in the first year of the intervention delivery; (2) to improve our understanding of the moderating factors and modifications affecting intervention fidelity; and (3) to explore the feasibility of the PReCaP fidelity assessment in view of the modifications.

**Methods:**

Based on the PReCaP description we developed a fidelity instrument incorporating nineteen (n=19) intervention components. A combination of data collection methods was utilized, i.e. data collection from patient records and individual Goal Attainment Scaling care plans, in-depth interviews with stakeholders, and non-participant observations. Descriptive analysis was performed to obtain levels of fidelity of each of the nineteen PReCaP components. Moderating factors were identified by using the Conceptual Framework for Implementation Fidelity.

**Results:**

Ten of the nineteen intervention components were always or often delivered to the group of twenty elderly patients. Moderating factors, such as facilitating strategies and context were useful in explaining the non- or low-adherence of particular intervention components.

**Conclusions:**

Fidelity assessment was carried out to evaluate the adherence to the PReCaP in the Vlietland Ziekenhuis in the Netherlands. Given that the fidelity was assessed in the first year of PReCaP implementation it was commendable that ten of the nineteen intervention components were performed always or often. The adequate delivery of the intervention components strongly depended on various moderating factors. Since the intervention is still developing and undergoing continuous modifications, it has been concluded that the fidelity criteria should evolve with the modified intervention. Furthermore, repeated intervention fidelity assessments will be necessary to ensure a valid and reliable fidelity assessment of the PReCaP.

**Trial registration:**

The Netherlands National Trial Register: NTR2317

## Background

Geriatric care interventions are complex, dynamic, and patient-oriented in order to be sensitive and responsive to the unique characteristics of the elderly patient. This approach is essential to ensure clinically meaningful and relevant interventions. Yet, it is this approach, that makes it challenging to establish the reliable and competent delivery of geriatric interventions, in the literature referred to as intervention fidelity [1] or implementation fidelity [2,3]. Only an appropriate evaluation of the intervention fidelity can assess its contribution to the effects on performance.

In outcome research intervention fidelity has been described as the confirmation that the manipulation of the independent variable occurred as planned, which enables researchers to (1) determine how adequately a program model has been implemented; (2) assess conformity with prescribed components and absence of non-prescribed components; and (3) provide assurances to policy-makers that services are being implemented as intended and are reaching the target audience [4]. Without documentation and/or measurement of adherence to the intended intervention, it is impossible to determine whether unsuccessful outcomes reflect failure of the intervention or failure to implement the intervention as intended, or even the influence of moderating factors [5,6]. Yet, intervention fidelity is seldom measured in geriatric health services research.

Intervention fidelity has two core components: protocol adherence and competence. Adherence is the most basic and entails the extent to which the interventionists’ behavior conforms to the intervention protocol in terms of content, frequency and duration [7]. The competence component is more complex and focuses on the interventionist’s skillfulness in the delivery of the intervention. Both components are necessary for an inference of validity, because an interventionist’s ability to engage participants can affect whether offered interventions are used and have adequate opportunities to produce change [1]. Fidelity assessment can address the following issues that may arise during the implementation of any intervention:

● Content

 ● Characteristics (e.g. complexity, resources and equipment, number of interventionists, time needed for intervention components)

 ● Discriminability of evaluated components

 ● Overlap of components (e.g. was there contamination of the independent variable)

● Process

 ● Description of the implementation strategy

 ● Implementation of components in accordance with the protocol

 ● Acceptance of the intervention by the interventionists and the patients

 ● Use of effective communication skills and behaviours

 ● Variation in the intervention delivery across departments

 ● Change of the intervention delivery across time (e.g. did the components weaken as they were delivered over the course of time?)

 ● Change of the intervention delivery throughout the course of the study period (e.g. did the interventionists drift as they delivered the intervention?)

 ● Variance in the delivery of a particular component to different types of patients (e.g. did more severe patients receive different components than less severe ones?)

● Outcomes

 ● Association between particular components and good (or poor) outcomes

 ● Association between intervention ‘purity’ and better outcomes

Traditional outcome research often assumed that the provision of a detailed description of the intervention (e.g. in the form of a manual) was enough to ensure the adequate implementation in the field. Consequently, it was assumed that patients received the interventions they were supposed to receive as designed [8]. Studies in the early eighties however, showed that this belief was flawed [9,10]. Recognition of the importance of establishing fidelity emerged from the difficult, if not impossible comparison of effectiveness, the growing emphasis on accountability, and the gap between research and practice [8]. Nowadays the verification of intervention fidelity is considered an essential facet of outcome research [1,7,8,11].

An important issue to consider in fidelity assessment is the influence of moderating factors on the implementation of the intervention. According to Carroll’s Conceptual Framework for Implementation Fidelity, these factors include comprehensiveness of policy description, facilitation strategies, quality of delivery, and participant responsiveness [11]. Complex and vaguely described interventions are assumed to be more difficult to implement with high fidelity than simple interventions [12]. Suitable facilitating strategies include the provision of manuals, guidelines, training, monitoring and feedback for those delivering the intervention. Such strategies increase opportunities for higher and more standardized fidelity. Quality of delivery concerns ‘the extent to which a provider approaches a theoretical ideal in terms of delivering program content’ [2]. Participant responsiveness refers to those delivering as well as those receiving the intervention, thereby depending on its acceptance and acceptability [11,13]. Carroll’s framework has been modified by the addition of two moderating factors, i.e. context and participant recruitment [14]. Yet, there is little empirical research on the factors that moderate intervention fidelity, as most studies have focused solely on adherence [2,11].

Despite calls for process evaluation as a necessary component of randomized clinical trials in the field of geriatric care [7,15], studies assessing fidelity of geriatric interventions lag far behind the number of effect studies in this field. This has occurred for several reasons, one of the foremost being that compared with the effect studies, it is difficult to specify the independent treatment variable and deliver it in a uniform way [16]. The nature of geriatric care interventions is that they are complex, multidisciplinary and dynamic. These characteristics provide the essential strength as well as weakness, as they increase the risk of variance, due to the number of intervention components, the multiple interventionists, and the heterogeneity of the group of geriatric patients [6,17]. Furthermore, developing intervention fidelity rating systems, train intervention fidelity raters, and establish reliability of ratings is difficult, expensive, and time-consuming [18,19].

### Case study: The Prevention and Reactivation Care Program

Hospital related functional decline in older patients is an underestimated problem. Thirty-five percent of 70-year old patients experience functional decline during hospital admission in comparison with pre-illness baseline [20]. This percentage increases considerably with age. To address this issue, the Argos Zorggroep in cooperation with the Vlietland Ziekenhuis in The Netherlands has implemented the pilot Prevention and Reactivation Care Program (PReCaP) in early 2010. The PReCaP entails an innovative program aimed at reducing hospital related functional decline among elderly patients by offering interventions that are multidisciplinary, integrated and goal oriented at the physical, social, and psychological domains of functional decline. The intervention incorporates five distinctive elements: (1) Early identification of elderly patients with a high risk of functional decline, and if necessary followed by the start of the reactivation treatment within 48 hours after hospital admission; (2) Intensive follow-up treatment for a selected patient group at the Prevention and Reactivation Centre (PRC); (3) Availability of multidisciplinary geriatric expertise; (4) Provision of support and consultation of relevant professionals to informal caregivers; and (5) Intensive follow-up throughout the entire chain of care by a casemanager with geriatric expertise [21,22]. Given the multidisciplinary approach and the complexity of the PReCaP, it is anticipated that deviations from the PReCaP will occur to tailor the program to the local circumstances and resources and to meet the social and cultural needs of patients. Hence, as part of a larger evaluation study [22], an intervention fidelity assessment was carried out in the hospital phase of the PReCaP from November 2010 to October 2011. The study has the following objectives:

1. To systematically assess intervention fidelity of the hospital phase of the Prevention and Reactivation Care Program;

2. To improve our understanding of the moderating factors and modifications affecting intervention fidelity;

3. To explore the feasibility of the PReCaP fidelity assessment in view of the modifications.

It is anticipated that the results of the intervention fidelity study will assist in strengthening the impact of the evaluation study [22]. Moreover, the results will be used to further refine the PReCaP and adapt for other hospital settings where elderly patients at risk for functional decline can benefit from the PReCaP intervention and philosophy. The PReCaP also incorporates six follow-up treatment routes for reactivation after hospital discharge [21,22], which will be assessed for fidelity in a separate study.

## Methods

A literature review was conducted to determine procedures for fidelity assessment utilized by other researchers, which resulted in a synthesis of methodologies across studies [4,23,24]. In order to maximize the validity of the results, we used a combination of methods, i.e. data collection from patient records; in-depth interviews with key stakeholders to reflect on the results and to identify moderating factors influencing intervention fidelity; and non-participant observations. To establish the fidelity criteria we followed three major steps, described by Teague, Bond & Drake [24]:

### Step 1: identification of the essential intervention components

The various components of the PReCaP were outlined in the description of the intervention, based on a me-thod proposed by Perera, Heneghan & Yudkin [25]. This method of graphically depicting randomized trials of complex interventions is developed to specify the different components of the intervention, to establish the time in which components are delivered and to define the differences between intervention arms. The description of the intervention was utilized to identify 16 essential and 3 conditional components, thereby ensuring consistency with the intervention and its theoretical underpinnings (Table 1), [17,21].

**Table 1:** PReCaP: intervention fidelity and moderating factors

	**Adherence extent**	**PReCaP Core Staff**	**Moderating factor**
**Day 1**			
1. Identification of patient at risk within 48 hours after admission	Always	Research nurse	Recruitment
	(Performed in time-often)		Context
	(Performed later-sometimes)		
2. Assessment of risk factors for functional decline	Always	Research nurse	
	(Performed in time-sometimes)		
	(Performed later-sometimes)		
3. Consultation with patient and relatives to discuss vulnerability and risk factors	Often	Casemanager or geriatric nurse	Participant responsiveness
**Day 2**			
4. Patient discussed in biweekly Multidisciplinary Team Meeting (MTM)	Always	Geriatrician	Context
		Geriatric nurse	
		Nurse practitioner	
		Social worker	
		Transfer nurse	
		Casemanager	
Design GAS care plan including advice for additional treatment aimed at functional preservation	Always	Geriatrician	Facilitation strategies
		Geriatric nurse	
		Nurse practitioner	
		Social worker	
		Transfer nurse	
		Casemanager	
**Day 3 – 5**			
5. Consultation following MTM	Often	Casemanager	
		Geriatric nurse	
		Transfer nurse	
		Geriatrician	
6. Consultation with patient and relatives to discuss vulnerability and risk factors	Seldom	Casemanager and/or geriatric nurse	Comprehensiveness of policy description
7. Interdisciplinary consultation following MTM	Often		
Psychiatrist, psychiatrist, occupational therapist, dietician			
**Day 6 – 7**			
8. Support and provide treatment to informal caregiver (conditional)	Never	Social worker	Participant responsiveness
		Psychologist	
9. Medication use review by pharmacist	Never		Context
10. Treatment by PReCaP Recovery Team (conditional)			
Casemanager	Sometimes		
Art therapist	Seldom		
**Day 8**			
11. MTM - Review prognosis and discharge destination (in some cases register patient at hospital replacement care facility)	Sometimes	Geriatrician	
		Geriatric nurse	
		Nurse practitioner	
		Social worker	
		Transfer nurse	
		Casemanager	
12. Weekly telephone consultation informal caregiver	Always	Casemanager	
13. Consultation with patient and relatives to discuss vulnerability and risk factors	Seldom	Casemanager and/or geriatric nurse	Comprehensiveness of policy description
14. Hand out flyer ‘PReCaP Recovery Team’ to patient	Always	Casemanager	Participant responsiveness
**Day 9**			
15. Execution PReCaP care plan			
Physiotherapist, dietician, occupational therapist	Sometimes		
**Before day 12**			
16. Exit interview with patient and informal caregiver	Sometimes	Casemanager or transfer nurse	Context
17. Send flyer ‘Prevention and Reactivation Centre’ to informal care giver’s home address (if transfer to PRC) (conditional)	Always	Casemanager	Participant responsiveness
18. Handover GAS care plan to physician hospital replacement care facility	Sometimes	Casemanager or geriatrician	

Never = 0%; Seldom = 1-33%; Sometimes = 34-66%; Often = 67-99%; Always = 100%.

### Step 2: construction of scale items

Fidelity criteria often include specification of the four adherence components, i.e. content, frequency, duration, coverage [7]. Kelly et al. [26] suggest additional specifications of roles, qualifications, and activities of staff. Yet, we were unable to detect any measure of timeliness in the literature. Given that the identification of the vulnerable elderly patient, the assessment of risk factors for functional decline, and the start of the reactivation treatment should be performed within 48 hours after admission, timeliness is considered to be of vital importance for these components [21]. Hence, we constructed dichotomous scales to capture the timing of these intervention components, i.e. (1) performed in time and (2) performed later. We performed a preliminary test of the intervention fidelity instrument to ensure that the data collection methods would work, and data would be collected appropriately. Therefore, we screened two patient files, which showed that the intervention fidelity instrument could be used to suitably collect the necessary data. We added an open section in the intervention fidelity instrument to allow for additional data, which could not be captured by means of the items in the instrument.

### Step 3: measuring fidelity

Fidelity data were collected by means of document analysis in the Vlietland Ziekenhuis, Schiedam, The Netherlands. Taking a pragmatic approach [27], 20 patient records and individual Goal Attainment Scaling (GAS) care plans from patients hospitalized in the period November 2010 - October 2011 were randomly selected from the research data base and screened for evidence of delivered PReCaP components. Due to organizational constraints, the PReCaP was implemented only in the geriatric, cardiology and internal medicine unit, which limited the data collection to these particular units. Five interviews were conducted with the casemanagers and the program leader to reflect on the results of the document analysis, and to identify moderating factors for non-adherences and obscurities in the data concerning the actual delivery of the intervention components. The casemanagers were selected to be interviewed, because of their central role in the PReCaP. The interviews lasted approximately 60 minutes. The first and second author of this paper (AJBMdV and JDHvW) undertook non-participant observations during five multi-disciplinary team meetings (MTMs) in the Vlietland Ziekenhuis.

### Step 4: assessing reliability and validity of the fidelity criteria

The components of the intervention fidelity instrument were established to ensure an accurate reflection of the PReCaP. The PReCaP Recovery Team, consisting of the program director/psycho geriatrician, program leader, and casemanagers with geriatric expertise critically reviewed the intervention fidelity instrument to ensure construct validity [3].

### Analysis

Descriptive analysis in IBM SPSS Statistics 19 was performed to obtain levels of fidelity for each of the 19 PReCaP components. Fidelity levels were determined as follows: never (0%), seldom (1-33%), sometimes (34-66%), often (67-99%), and always (100%).

The interviews were transcribed and analyzed using framework analysis [28]. Four steps were performed to ensure the quality of the analysis [29]: (1) Reading all the material to form an overall impression; (2) Identifying and coding the moderating factors (and their potential interrelationships) as proposed by Carroll [11] and Hasson [7]: intervention complexity, facilitation strategies, quality of delivery, participant responsiveness, context, and participant recruitment; (3) Condensing and summarizing the contents of each of the groups; and (4) Generalizing the description and contents reflecting the moderating factors. Field notes from observations were analyzed for emerging themes independently from the interviews. Relevant themes were then assessed against findings from interviews, with special interest on observations that could illuminate accounts that participants gave in interviews. The study was approved by the Medical Ethics Committee of the Erasmus Medical Centre, Rotterdam, The Netherlands under protocol number MEC2011-041.

## Results

Patient records and individual GAS care plans were assessed for evidence of delivered PReCaP components. The patients’ demographics are presented in Table 2. The admission diagnosis incorporated a wide range, and often a combination, of diseases and medical conditions, including lethargy, fever, abdominal discomfort, pneumonia, hepatic alcohol-abuse, hip fracture, pulmonary embolism and cardiac asthma. The wide range of medical conditions evidently shows the heterogeneity of the group of elderly patients, and hence the requirement for multidisciplinary and integrated interventions.

**Table 2:** Demographics (n=20)

	**n**	**%**
**Gender**		
Male	8	40
Female	12	60
**Age group**		
65–69	2	10
70–74	2	10
75–79	4	20
80–84	3	15
85–89	5	25
90–94	3	15
**Hospital days**		
1–4	1	5
5–9	7	35
10–14	7	35
15–19	4	20
20–24	1	5
**Unit**		
Geriatrics	8	40
Internal Medicine	8	40
Cardiology	4	20
**Discharge destination**		
Prevention and Reactivation Centre	5	25
Nursing home	1	5
Retirement home	3	15
Home	11	55

### Adherence

The PReCaP encompassed 19 components during the hospital phase (Table 1). Of these, ten components were always or often delivered. These included ‘Identification of patient at risk within 48 hours after admission’ (90% in time; 10% later) and ‘Assessment of risk factors for functional decline’ (55% in time; 45% later). ‘Consultation with patient and relatives to discuss vulnerability and risk factors’ took place in 90 per cent of the cases. Yet, the scheduled ‘Consultation with patient and relatives to discuss vulnerability and risk factors’ on day 3–5 took place in only one case. Bi-weekly MTMs were held in accordance with the description of the PReCaP, during which the team members (geriatrician, geriatric nurse, nurse practitioner, social worker, transfer nurse, and casemanager) designed the GAS care plan, including advice for reactivation treatment aimed at functional preservation. Following the advice of the MTM, the casemanager with geriatric expertise performed a consultation with the elderly patients in 75 per cent of the cases, in some cases concurrently with the geriatric nurse, the transfer nurse, and the geriatrician. In addition, the advice of the MTM resulted often in interdisciplinary consultations by the psychiatrist, physiotherapist, occupational therapist, and dietician on day 3–5. The component not delivered according to the description of the PReCaP concerned the conditional component ‘Support and provide treatment to informal caregiver by social worker or psychologist’. Furthermore, non-adherence related to ‘Medication use review by pharmacist’ due to time and organizational constraints at the time of the fidelity assessment. The PReCaP Recovery Team treatment involves conditional input (i.e. under certain circumstances) from various disciplines, including the casemanager, physiotherapist, occupational therapist, speech therapist, dietician, psychologist, pharmacist, nursing home physician, social worker, art therapist, and behavioral therapist. The results revealed that of the twenty patients, one patient received dedicated treatment from the art therapist and nine patients received treatment from the casemanager. The PReCaP prescribes on day 8 ‘MTM - Review prognosis and discharge destination (in some cases register patient at hospital replacement care facility)’. This component was documented in either the patient record or the individual GAS care plan in 65 per cent of the cases. ‘Consult with patient and relatives to discuss vulnerability and risk factors’ by either the casemanager or the geriatric nurse was reported in just two cases. All patients were handed out the flyer ‘PReCaP Recovery Team’, which introduces the team, comprising of a casemanager with geriatric expertise, psycho geriatrician (and if indicated a dietician, behavioral therapist, or art therapist), and presents an overview of the treatment. ‘Exit interview with patient and informal caregiver’ was reported in half of the cases, and if patients were transferred to the PRC, the flyer ‘Prevention and Reactivation Centre’ was always sent to the informal caregiver’s home address. For 55% of the cases (n=11), the casemanager or geriatrician was reported to have handed over the GAS care plan to the physician in the hospital replacement care facility.

### Moderating factors

#### Recruitment

Although the components ‘identification of patient at risk within 48 hours after admission’ and ‘assessment of risk factors for functional decline’ were performed according to the intervention description (Table 1), the research nurses expressed their concern about the non-participation among the group of extremely ill and vulnerable patients. The research nurses observed that the frailest patients declined to participate due to their physical and/or mental condition at the time of hospital admission.

#### Facilitation strategies

The provision of guidelines and manuals, monitoring and feedback are known strategies to optimize and standardize implementation fidelity, i.e. to ensure that all interventionists receive the same training and support, aimed at a uniform delivery of the intervention [30]. Although a description of the PReCaP was available for the casemanagers, a comprehensive protocol systematically outlining the components and timeline of the PReCaP was lacking. In particular, the nurses on the ward expressed their concern about the paucity of information regarding the implementation of the PReCaP, which evidently demonstrates the complex interrelationship between facilitation strategies and participant responsiveness [11]. In response to the articulated need for support, the casemanagers and project leader are preparing an intervention protocol.

“*The PReCaP intervention is dynamic and continuously improving*. *When we started in 2010 we utilized the description of the PReCaP*, *which specifies the different components of the intervention*, *as a guide for the intervention activities*. *Based on this description*, *we are now preparing a protocol according to the standards of the Dutch Harmonization Quality Assessment in Healthcare* [*Harmonisatie Kwaliteitsbeoordeling in de Zorgsector*]. *We believe that there needs to be a protocol to ensure continuity of the PReCaP*, *but at the same time allows adding or omitting intervention components*.” (Casemanager)

Since December 2011, the casemanagers use issue reports to explore unplanned implementation issues (i.e. incidents or deviations from the PReCaP description), and to prevent these issues from happening in the future. These reports incorporate a description of the issue and the situation, the impact on the patient, the level of priority, and the planned action. The PReCaP Recovery Team, including program director/geriatrician, program leader, and casemanagers convenes four-weekly to discuss the quality of the intervention and to address implementation issues if necessary. Below is an outline of an issue report.

● Issue: GAS care plans are not designed during MTM

● Description: According to PReCaP description GAS care plans should be designed during MTM

● Attending team members refuse to cooperate due to time constraints and lack of motivation

● Impact: Goals and advice for reactivation treatment (aimed at functional preservation) are not formulated. Reactivation treatment does not start within the set time frame of 48 hours after hospital admission

● Priority: High

● Action:

  1. During the next MTM the casemanagers emphasize the importance of the timely design of the PReCaP care plan to team members to enable timely start of the reactivation treatment

  2. During the MTM the team members draft the GAS care plan on paper. Casemanagers enter the GAS care plan draft in the GAS data base on the same day

● Deadline: four weeks

#### Context

Contextual factors had an immediate effect on the intended implementation of the PReCaP. Despite earlier agreements, the management of the Vlietland Ziekenhuis allowed only three of the ten hospital units to participate in the intervention. This decision undoubtedly affected the recruitment and the case mix of the participants in the PReCaP, which shows the complex interrelationship between the context and recruitment [11]. Furthermore, due to a reorganization procedure, the Internal Medicine and Oncology ward merged. It is highly likely that this merge impacted on the case mix of the participants in the PReCaP, due to the increased number of oncology patients.

According to the description of the PReCaP, the research nurse performs the components 1 and 2 (i.e. ‘Identification of patient at risk within 48 hours after admission’ and ‘Assessment of risk factors for functional decline’). Therefore, permission was obtained from the Vlietland Ziekenhuis to access the admissions department database, enabling identification of the patients at risk (65 years or older and admitted for at least two days) within 48 hours after admission.

Given that polypharmacy is a known risk factor for elderly patients [31], ‘review of the elderly patient’s medication by the pharmacist’ entails an essential component of the PReCaP. Yet, this component had not been implemented due to time and organizational constraints, i.e. the rapid patient flow in the hospital phase. At the time of writing, this component had been incorporated in the PReCaP admission protocol (TJEM Bakker, personal communication, June 15, 2012).

The PReCaP prescribes the casemanager to conduct the exit interview with the patient and the informal caregiver. However, it is the Vlietland Ziekenhuis policy that the transfer nurses perform all exit interviews to ensure coordination and continuity of the discharge procedures. Therefore, this component was modified to comply with hospital policy.

#### Quality of delivery

The quality of delivery concerns the extent to which an intervention is delivered in a way appropriate to achieving what was intended [11]. In studies evaluating fidelity, the provision of training and support to those delivering the intervention has been considered to contribute to optimization of the quality of the intervention delivery.

The Vlietland Ziekenhuis and Argos Zorggroep have collaboratively developed a geriatric training program for registered nurses and nurse practitioners. Prior to the commencement of the PReCaP in 2010, fifty Registered Nurses in the Vlietland Ziekenhuis have undertaken the particular training program. In addition, three clinical geriatricians were employed, nursing home physicians have undertaken gerontology-rehabilitation training, and psychologists have undertaken training in system therapy.

#### Participant responsiveness

Participant responsiveness refers to how well participants respond to, or are engaged by, an intervention. It also involves judgments by the interventionists about the outcomes and relevance of an intervention [11]. The PReCaP prescribed consultation of the patient and relatives to discuss vulnerability and risk factors on Day 1 by either the casemanager or the geriatric nurse. In most cases the casemanager performed this task, but not before Day 2 in order to prevent mental overload of the patient on the day of hospital admission (due to e.g. doctor’s consultations, research nurse visit, blood tests, X-rays). The casemanager combined this visit with obtaining data via heteroanamnesis from the informal caregiver.

“*Our aim is to prevent mental overload of the patient*. *For example*, *when you present the various follow*-*up treatment routes one can see the patient not taking it in or becoming stressed and confused*. *Therefore*, *we have agreed not to visit the patient on the same day as the research nurse*.” (Casemanager)

Since mid-2011 the Geriatrics unit team leader and the PRC nursing home physician participated in the MTM. In particular, the extra-mural vision of the nursing home physician was considered to be of added value, in particular with regard to the post-discharge period.

“*Some patients just want to go home despite the known risk factors*. *During the MTM the nursing home physician clarifies the reasons why it is impossible for the particular patient to go home*, *and that we should advice continued treatment for both the patient and the informal care giver*. *There should be minimum conditions before a patient returns home* …. *for example*, *that someone can stand on his own two feet*. *The team members accept the input from the nursing home physician*.” (Casemanager)

The PReCaP description prescribed that the casemanagers handed out information flyers to the eligible patients for the Prevention and Reactivation Centre. Yet, this appeared to be ineffective, and an alternative option to inform the patient and the informal care giver was chosen.

*Following the MTM*’*s advice for follow*-*up treatment at the PRC* (*component 12*) *we used to hand out the flyer* ‘*Prevention and Reactivation Centre*’ *to the patient*, *but this appeared to be too much information for the patients to process*. *Therefore*, *we now mail the flyer to the informal care giver*’*s home address*. *We have to be very much aware of the fact that we are dealing with frail elderly patients*.”(Casemanager)

## Discussion

The Prevention and Reactivation Care Program (PReCaP) was developed as a means to reduce hospital related functional decline among elderly patients by offering interventions that are multidisciplinary, integrated and goal-oriented at the physical, social, and psychological domains of functional decline. Evaluation of the PReCaP, currently underway, is expected to determine the extent to which the program leads to improved geriatric care as well as cost reduction in comparison to current geriatric care in The Netherlands [22].

Fidelity assessment is considered essential in order to maintain internal validity and to ensure a fair comparison of the results between the intervention and control settings. Results without a fidelity check may be due to an effective intervention or contamination from other interventions [6]. The issue of intervention fidelity also pertains to external validity. In order for a particular intervention to be adopted by other hospital settings, sufficient information about the method, fidelity, and effectiveness of all relevant intervention components is essential for effective implementation [1,4,32].

Studies assessing fidelity of geriatric care interventions are rare. Thus far, most fidelity studies were performed in the field of psychiatric rehabilitation (Carroll et al. 2000, Bond et al. 2000, Song, Happ & Sandelowski 2010) and prevention programs in schools (Dane, Schneider 1998). Despite calls for process evaluation as a required component of randomized clinical trials in the field of geriatric care (Buurman 2011), fidelity studies are lacking in the field of geriatric care interventions.

This paper presents the results of a fidelity assessment, carried out to evaluate the adherence to the PReCaP (hospital phase) in the Vlietland Ziekenhuis in The Netherlands and to improve our understanding of the moderating factors affecting intervention fidelity. The results showed that ten of the nineteen intervention components were always or often delivered to the group of twenty elderly patients. In particular, the identification of the patient at risk and the assessment of risk factors were performed in accordance with the PReCaP. This was in contrast with a study performed in the Emergency Department, where participant recruitment was problematic due to time constraints in conducting the geriatric assessment, and difficulties finding individuals that fulfilled the inclusion criteria [7].

Multi-disciplinary team meetings (MTMs), inter-disciplinary consultations and treatments were largely conducted according to the PReCaP. Yet, it is not clear whether the latter two were the result of the PReCaP intervention (or more specifically the MTM component) or the specialist’s orders, an issue known as the ‘essential but not unique’ components of an intervention [1,16]. The GAS care plan, including advice for additional treatment aimed at functional preservation - an essential component of the PReCaP intervention - was designed in all cases either in time or delayed. The delay in designing the GAS care plan may have been caused by the fact that the MTM convened twice a week, i.e. on Monday and Thursday. When a patient was admitted on Friday, he or she would not be discussed in the MTM until the next Monday, which is obviously after the ‘48 hours after admission’ time slot. The casemanagers ensured issuance of the information flyers about the PReCaP Recovery Team and the PRC by sending the flyers to the home address of the informal care giver, after a PRC advice had been issued. A small number of components was not delivered due to various factors. For example, consultation with patient and relatives to discuss vulnerability and risk factors on Day 3–5 and Day 8 was performed in respectively one and two cases. This may have been caused by the fact that the particular consultations are not considered to form an essential component of the intervention. Another explanation for the low fidelity rate may have been the fact that a large proportion of cardiology patients were short-term admissions and discharged after 2–4 days. The intervention component ‘Medication use review by pharmacist’ has not been implemented yet, due to time and organizational constraints, i.e. the rapid patient flow in the hospital phase. The weekly telephone consultation for the informal caregiver was performed in only 10 percent of the cases. It is highly likely that underreporting has caused the low fidelity rate for this component, since the casemanagers claimed that they contacted the informal care givers of hospital patients on a weekly basis. Underreporting may also have played a moderating role in the adherence rates of the components ‘Exit interview with patient and informal caregiver’ and ‘Handover GAS care plan to physician hospital replacement care facility’, which were reported as having been performed for just 55 percent of the patients, because the casemanager assured that these components - without fail - were performed. These results show the importance of collecting data from multiple sources in fidelity assessment to minimize reporting bias. Yet, data collection from multiple sources can complicate the understanding and interpretation of the results [4]. Therefore, differences in perspective (reporting staff, casemanagers, MTM members) and response characteristics (i.e. patient records, interviews, and observations) may require examination of reliability across the researchers by calculating the correlation (e.g. Cohen’s kappa) between the perspectives and response characteristics in future measurements.

The absence of a comprehensive protocol may have caused reduced fidelity rates due to interventionists’ (e.g. nurses in the ward) unfamiliarity with the PReCaP components. A well written comprehensive protocol outlines the intervention and presents the theory, the goals, and the strategies for achieving these goals. Moreover, a protocol provides objective means for comparing interventions and facilitating transfer from research to practice. Therefore, utilizing a comprehensive protocol will lead to greater consistency and precision in the intervention delivery and enhanced internal validity [1]. Moreover, a comprehensive protocol establishes the balance between standardization to support adherence and internal validity, and flexibility to support competence and external validity [1].

The interview results demonstrated that the PReCaP is still developing and undergoing continuous modifications. For example, in contrast with the initial PReCaP description, the casemanagers do not visit the patient until Day 2 to prevent mental overload, and since mid-2011 additional professionals (the Geriatrics unit team leader and the PRC nursing home physician) participate in the MTM to enhance the multidisciplinary input. Evidence suggests that programs are usually not static and often undergo substantial changes over time [4].

There has been a long-standing controversy between schools of thought that advocate for exact replications of effective program models versus the need to adapt models to local conditions to maximize efficiency as well as local ownership, also referred to in the literature as reinvention [33]. In this study, it was identified that the intervention component ‘Medication use review by pharmacist’ had not been implemented due to time and organizational constraints. Although deemed undesirable, it represents a good example of how a ‘potentially’ essential intervention component can be affected by local conditions. It is generally agreed that programs with higher fidelity to efficacious models produce superior outcomes [34,35]. Szulanski and Winter [36] state that a best practice should be copied as closely as possible, in minute detail, and that adapting a successful template is a mistake. On the other hand, there is often a legitimate need to tailor a program to local circumstances and resources and to the social and cultural needs of the target population [37-39]. For example, patients in different locations have different strengths and needs and health service organizations have different goals and objectives. Taking a theoretical approach, adaptations to local circumstances are seen as appropriate as long as they do not contradict the underlying program theory. In this approach, the program provides a ‘cognitive blueprint’ for action [40]. Staff members are not expected to follow process protocols exactly, but rather, according to their own judgments of what fits with the patient characteristics and context and the program theory. This approach is congruent with research about professionals being more engaged, motivated, and effective when they feel they are exercising their judgment and expertise [41]. Some fidelity opponents fear that too much emphasis on fidelity will stifle creativity and promote a treatment philosophy of ‘one size fits all’ (Bond et al., 2000). Interventions can be defined in terms of essential elements likely to be responsible for effectiveness. These essential elements cannot be changed without fundamentally changing the intervention, whereas other characteristics may be modified without altering effectiveness [26]. In view of fidelity assessment, this implies that determination of the essential components is of vital importance. Furthermore, fidelity criteria should evolve concurrently with the modifications of the intervention in order to obtain valid measurements.

Although the PReCaP fidelity assessment was demonstrated to be a feasible measurement instrument, it also showed the need for further articulation of the criteria for inclusion of the conditional intervention components (e.g. ‘Support and provide treatment to informal caregiver by social worker or psychologist). Other components (e.g. ‘Consultation with patients and relatives to discuss vulnerability and risk factors’), though considered essential, did not apply to some patients. In addition, a component might be deemed ‘essential’, but its frequency or dose might be open to modification. Therefore, refining and further articulation of the criteria will be necessary to ensure ecological validity of the instrument.

The current PReCaP instrument includes two components which are - at least implicitly – double-barreled, i.e. ‘Identification of patient at risk within 48 hours after admission’ and ‘Assessment of risk factors for functional decline’ by specifying both activity and timing in its label. In rating the component, it was broken apart to allow credit for adherence independent of time followed by categorization of the timing of delivery. Although it did not present a problem at this preliminary stage, feasible application, including quantification of assessment results, may require expanding the measure to put adherence and timing in separate items.

In this measurement, we have based the assumption of quality of intervention delivery on the pre- and in-service training. Yet, a study evaluating fidelity of community-based treatments for people with serious mental illness suggested that introductory training, supplemented by the use of written materials and phone-based consultation is not an adequate proxy for the assumption of quality of intervention delivery [42]. Therefore, we anticipate assessing the quality aspect more directly in future stages of the development of the PReCaP instrument.

As part of the larger evaluation study [22] we anticipate comparing the care process for elderly patients in the Vlietland Hosptial, Schiedam (intervention setting) and the Sint Franciscus Gasthuis Rotterdam and the Ruwaard van Putten Ziekenhuis, Spijkenisse (both control settings). However, given that the latter two hospitals provide elderly care as usual, it will not be feasible to carry out a fidelity assessment using the PReCaP instrument in the control settings. Instead, we will collect data from patient records using process indicators, perform in-depth interviews with professionals and carry out non-participant observations.

### Methodological limitations

The design of the intervention fidelity instrument was critically reviewed by the program director/psycho geriatrician, program leader, and casemanagers to ensure the construct validity of the instrument. Although expert panel consensus has been used in various phases of the development and use of fidelity instruments [3,24,43], several issues have been described [4]. The predictive utility of expert opinion is considered quite low. Schemes for grading levels of evidence for interventions place expert opinion lowest in the hierarchy of knowledge [44]. Another issue concerns the availability of experts and their credibility, as there are different perspectives on what may constitute expertise. To address this issue, we included multiple perspectives in the review panel.

The results suggest that intervention fidelity assessment is associated with potential under-reporting, a view also expressed by Carroll et al. [11], who called for improving the reporting of complex interventions to identify and address potential sources of heterogeneity in implementation. It is highly likely that intervention fidelity research will benefit from triangulation by facilitating the validation of the data through cross verification from more than two sources. Hence, it is acknowledged that - in addition to the data collection from patient records, key stakeholders interviews, and non-participant observations - surveying patients and informal care givers, although potentially an ‘unreliable’ source, may have contributed to the validity of the results.

Another issue to consider is the potential effect of the fidelity assessment on the intervention, also known as the police car effect [45]. Interventionists’ awareness that the service is being evaluated can change their behaviour, i.e. they may perform better or worse than they would otherwise do, because they feel not only that they are under scrutiny, but that the results of the evaluation may have good or bad consequences for them. This is not a problem for developmental evaluations which aim to create or exploit a ‘Hawthorne effect’, but for experimental evaluations, such as the PReCaP evaluation, the effect may introduce a further variable, which is impossible to control for by ‘blinding’.

Although registered nurses, nurse practitioners, nursing home physicians, and psychologists have undertaken specialist training prior to the commencement of the PReCaP, the competence of the core interventionists was not specifically measured. Clearly, the provision of training has a moderating effect on the quality of the intervention delivery, which, in turn may affect the intervention fidelity. Yet, this relationship is more complex than may be captured in a simple correlation of training and fidelity numbers through the moderating role of the intervention complexity [11]. A simple intervention may require a small amount of training to achieve high fidelity rates. In contrast, a complex multidisciplinary intervention, such as the PReCaP, may require more intensive training to achieve similar fidelity outcomes. It is obvious that moderating factors, including recruitment, participant responsiveness, and context affect fidelity in a complex and interrelated way, which illustrates the challenges of assessing fidelity of a complex multidisciplinary intervention [7].

Finally, we acknowledge the narrow selection of the patient sample and setting. Therefore, we emphasize that the intervention fidelity results, although indicative for the PReCaP, are not necessarily generalizable to other settings and populations. Yet, the considerations with regard to the moderating factors and modifications affecting intervention fidelity can be viewed in the broader context.

## Conclusions

As part of a large evaluation study of a geriatric care intervention (PReCaP), a fidelity study was carried out in the first year of the program delivery to determine the extent to which each intervention component in the hospital phase was delivered as intended. A number of useful messages emerged from this study. Ten of the nineteen components were always or often delivered as set in the PReCaP description. The intervention adherence varied substantially per component due to a range of moderating factors, e.g. the lack of a comprehensiveness protocol resulted in interventionists not being familiar with the PReCaP intervention components. Furthermore, a number of intervention components were modified or added, which leaves the question to what extent the intervention objectives will be achieved by means of the modified intervention. Hence, it would be recommended to determine the essential intervention components and conduct repeated assessments of intervention fidelity over time in order to ensure a relevant and meaningful intervention.

## Abbreviations

MTM: Multi-disciplinary team meeting; PReCaP: Prevention and Reactivation Care Program; PRC: Prevention and Reactivation Centre; GAS: Goal Attainment Scaling.

## Competing interests

The authors declare that they have no competing interests.

## Authors’ contributions

AJBMdV prepared the manuscript and revised it for important intellectual content. TJEMB designed the intervention protocol, participated in the design of the evaluation study and revised the manuscript for important intellectual content. PLdV revised the manuscript for important intellectual content. JDHvW participated in the design of the evaluation study and revised the manuscript for important intellectual content. EWS participated in the design of the evaluation study and revised the manuscript for important intellectual content. JPM participated in the design of the evaluation study and revised the manuscript for important intellectual content. APN participated in the design of the evaluation study and revised the manuscript for important intellectual content. All authors read and approved the final manuscript.

## Pre-publication history

The pre-publication history for this paper can be accessed here:

http://www.biomedcentral.com/1472-6963/13/29/prepub
